# Repeatability of corneal and epithelial thickness measurements with anterior segment optical coherence tomography in keratoconus

**DOI:** 10.1371/journal.pone.0248350

**Published:** 2021-06-18

**Authors:** Ye Li, Akilesh Gokul, Charles McGhee, Mohammed Ziaei

**Affiliations:** Department of Ophthalmology, New Zealand National Eye Centre, Faculty of Medical and Health Sciences, University of Auckland, Auckland, New Zealand; Nicolaus Copernicus University, POLAND

## Abstract

**Purpose:**

To investigate the repeatability in corneal thickness (CT) and epithelial thickness (ET) measurements using spectral domain anterior segment optical coherence tomography (AS-OCT, REVO NX, Optopol) in keratoconus, and examine the effect of corneal crosslinking (CXL) on repeatability.

**Methods:**

A cross-sectional study of 259 eyes of 212 patients with keratoconus attending the corneal disease clinic at a university hospital tertiary referral center were enrolled. Two groups were analysed: eyes with no prior history of CXL (Group A) and eyes with prior CXL (Group B). Repeatability of measurements was assessed using the intraclass correlation coefficient (ICC) and coefficient of variation (CV).

**Results:**

In Group A, central corneal thickness (CCT) was 472.18 ± 45.41μm, and the ET was found to be the thinnest in the inferior-temporal aspect at 51.79 ± 5.97μm and thickest at the superior-nasal aspect at 56.07 ± 5.70μm. In Group B, CCT was 465.11± 42.28μm, and the ET was the thinnest at the inferior-temporal aspect at 50.63 ± 5.52μm and thickest at the superior aspect at 56.80 ± 6.39μm. When evaluating CT measurements, ICC was above 0.86 and 0.83 for Group A and Group B respectively. When evaluating ET measurements, ICC was above 0.82 for both groups. CXL had no statistically significant impact on the repeatability of measurements.

**Conclusions:**

AS-OCT provides repeatable CT and ET measurements in the central and peripheral cornea in patients with keratoconus. Repeatability is not affected by a history of CXL.

## Introduction

Keratoconus is a progressive, non-inflammatory corneal degeneration that leads to corneal thinning, irregular astigmatism and reduced visual acuity [1]. Corneal topography has long been the mainstay of diagnosis, staging, and progression analysis in this clinical entity, but in recent years, epithelial thickness (ET) maps have played an increasingly more prominent role in the early detection of subclinical cases [2]. Previous studies have shown that early detection of keratoconus with timely cornea crosslinking (CXL) can halt the progression of the disease, maintain or improve visual acuity and lead to a reduction in the number of patients requiring keratoplasty [3–7].

The corneal epithelium is a non-keratinized, stratified layer with a thickness of 48–53μm in healthy eyes, which has previously been shown to be moldable [8]. It has an asymmetrical thickness profile, being slightly thicker inferiorly and nasally [9]. In keratoconus, the corneal epithelium undergoes localized thinning over the cone, the steepest part of the cornea, surrounded by an annulus of epithelial thickening over the flatter regions of the cornea [10]. This epithelial compensation has been shown to mask the underlying irregularities of the corneal topography in ectasia, and previous reports have suggested that ET mapping can be a sensitive test for the diagnosis of early keratoconus [11, 12].

Spectral-domain anterior segment optical coherence tomography (AS-OCT) is widely used in anterior segment analysis. The AS-OCT is a non-contact device that creates cross-sections of the anterior segment, which can be generated into a three-dimensional structure to produce corneal thickness (CT) and ET measurements. AS-OCT is increasingly recognized as a sensitive tool for the diagnosis of keratoconus, which is comparable to Scheimpflug imaging [11]. AS-OCT is broadly classified into time-domain (TD-OCT) and Fourier-domain (FD-OCT), with the latter including spectral-domain (SD) and swept-source (SS) OCT [12]. REVO NX (Optopol Technology S.A, Zawiercie, Poland) is a new AS-OCT device that encompasses spectral-domain anterior and posterior segment optical coherence tomography with optical biometry technology [13].

Architectural distortion of the cornea in keratoconus coupled with surgical intervention, such as CXL, can affect the accuracy and repeatability of CT and ET measurements. While the repeatability of central corneal thickness (CCT) and thinnest corneal thickness (TCT) using AS-OCT is well reported in keratoconus [14], few studies have examined the repeatability of ET or the impact of CXL on repeatability.

This study aimed to investigate the repeatability of AS-OCT derived CT and ET measurements in patients with keratoconus with or without a prior history of CXL.

## Methods

This cross-sectional study enrolled patients with keratoconus attending the University of Auckland Cornea and External Eye Disease Service, Auckland District Health Board, Auckland, New Zealand.

Patients with keratoconus that was diagnosed based on clinical and tomographic findings were recruited. Those with corneal edema, severe dry eye, trauma or prior ocular surgery other than CXL were excluded. Contact lens wearers were asked to remove their contact lenses at least 24 hours prior to each examination. The analysis was split into eyes with no history of CXL (Group A) and eyes with a history of CXL (Group B). All patients had undergone CXL at least 3 months before their examination using an epithelium-off accelerated protocol described in detail elsewhere [3]. In brief, the central 8.0mm of the cornea was bathed in 20% ethanol for 20 seconds in a well, followed by epithelium removal using a blunt spatula and 4 minutes of ultraviolet-A exposure at 30 mW/cm^2^ and a total energy dose of 7.2 J/cm^2^ [3]. As keratoconus is an asymmetrical condition, with no predilection for either eye [15, 16], the right eye of patients was included in the analysis by default, and the left eye was included if any exclusion criteria applied to the right. The stage of keratoconus was established according to the Topographic Keratoconus Classification (TKC) system which classifies keratoconus into 5 stages: 0 (normal) to 4 (severe). Where the TKC value was between two stages, the severity was rounded up to the higher stage to allow for comparison of its effect on repeatability. Maximum keratometry (K_MAX_) and TKC were obtained with the Pentacam (Oculus, Wetzlar, Germany) [17].

The study was approved by the local Health and Disability Ethics Committee, a branch of the Ministry of Health in New Zealand. Written, informed consent was obtained from all patients after they voiced understanding of the purpose and the procedures of the study in accordance with the Declaration of Helsinki.

### Measurement instrument

The REVO NX is a non-contact device that incorporates optical biometry with anterior and posterior segment spectral-domain OCT. With an 830nm super-luminescent laser diode, this device scans 110,000 per second with a maximum scan depth of 2.4mm and a width of 16mm resulting in an axial resolution of 5μm and transverse resolution of 18μm [13].

**Fig 1:**
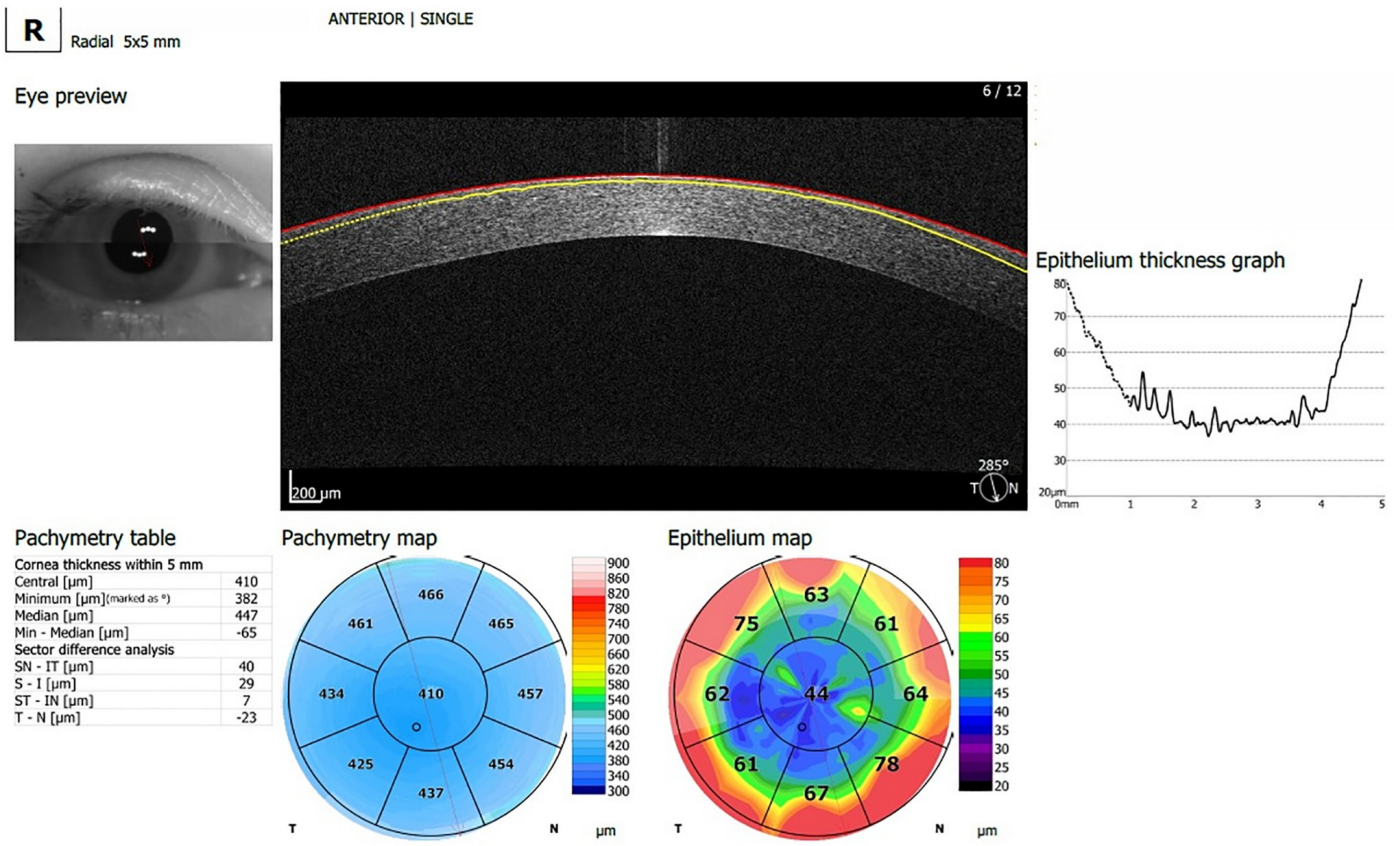
Corneal epithelial map produced by the REVO-NX AS-OCT.

Cross-sectional images are then created using Fourier-transformation and the ET maps in an 8mm diameter are automatically generated by the built-in software (version 5.5, Fig 1) [12].

### Patient assessment

A complete ocular assessment was performed. The REVO AS-OCT was calibrated upon initiation of the study. Each eye was consecutively scanned three times by one of two experienced investigators using manual capture in a dimmed room without pupil dilation, under identical lighting between 1:00 pm and 5:00 pm to reduce the effect of corneal swelling overnight [18]. Participants were asked to blink to allow corneal tear film coverage before measurements.

### Statistical analysis

Statistical analysis was conducted with SPSS (SPSS, IBM, Chicago, Illinois, USA). Kolmogorov-Smirnov test was used to assess for normality of distribution. Within-subject standard deviation (S_w_) was used to calculate precision (1.96 x S_w_) and repeatability (2.77 x S_w_) [19]. Coefficient of variation (CV) and intraclass correlation coefficient (ICC) were calculated from the three repeated scans. One-way ANOVA with Tukey’s post-hoc test was used to ascertain differences in repeatability between groups, as well as differences in repeatability between eyes of different disease severities. A *p*-value of <0.05 was deemed statistically significant.

## Results

### Demographics

Two hundred and fifty-nine eyes of 212 patients were included in the study (Table 1).

**Table 1:** Demographics of patients enrolled in the study.

Parameters		Value
**Patients (n)**		212
**Eyes (n)**		259
Group A		132 (42.7%)
	Right eye	100 (75.8%)
	Left eye	32 (24.2%)
Group B		127 (41.1%)
	Right eye	77 (60.6%)
	Left eye	50 (39.4%)
**Age (y, Mean ± SD)**		24.28±7.70
Group A		24.83±8.35
Group B		23.23±7.21
**Keratoconus stage**		
	1	55 (21.2%)
	1–2	20 (7.7%)
	2	56 (21.6%)
	2–3	29 (11.2%)
	3	63 (24.3%)
	3–4	32 (12.4%)
	4	4 (1.5%)
**K_MAX_ (D, Mean ± SD)**		56.13±8.50
Group A		54.88±9.21
Group B		57.42±7.52

K_MAX_, maximum keratometry

### CT and ET measurements

In both groups, inferior-temporal thinning and superior-nasal thickening was observed in CT and ET measurements. The CT of the eye shown in Fig 1 demonstrates this pattern. Tables 2 and 3 outline the CT and ET measurements in Groups A and B respectively.

**Table 2:** Measurements in Group A: Eyes without a history of crosslinking.

Sector	Mean ± SD (μm)	Within-Subject SD	Precision	Repeatability	CV (%)	ICC	ICC 95% CI
**Epithelial thickness**							
Superior	54.88 ± 5.89	3.35	6.57	9.29	4.66	0.89	0.86 to 0.92
Superior-temporal	54.77 ± 5.92	3.34	6.55	9.26	4.80	0.89	0.86 to 0.92
Temporal	52.06 ± 5.92	3.19	6.26	8.85	4.77	0.90	0.87 to 0.93
Inferior-temporal	51.79 ± 5.97	3.34	6.55	9.25	5.05	0.89	0.86 to 0.92
Inferior	53.26± 6.23	3.09	6.06	8.56	4.30	0.92	0.89 to 0.94
Inferior-nasal	55.05 ± 5.49	2.65	5.20	7.35	3.92	0.92	0.89 to 0.94
Nasal	56.03 ± 5.20	3.21	6.29	8.90	4.52	0.87	0.83 to 0.91
Superior-nasal	56.07 ± 5.70	4.15	8.13	11.50	5.46	0.82	0.76 to 0.87
Center	53.85 ± 6.12	2.30	4.51	6.38	3.20	0.95	0.94 to 0.97
**Corneal thickness**							
Superior	527.04 ± 37.66	18.03	35.33	49.93	1.98	0.92	0.89 to 0.94
Superior-temporal	507.24 ± 39.38	17.93	35.15	49.67	1.81	0.93	0.91 to 0.95
Temporal	479.35 ± 40.73	19.42	38.05	53.78	1.72	0.92	0.89 to 0.94
Inferior-temporal	468.99 ± 43.39	24.66	48.33	68.31	2.08	0.89	0.86 to 0.92
Inferior	483.24 ± 42.07	11.17	21.89	30.93	1.55	0.98	0.97 to 0.98
Inferior-nasal	505.06 ± 37.10	13.68	26.81	37.89	1.69	0.96	0.94 to 0.97
Nasal	520.97 ± 34.36	21.54	42.21	59.65	1.88	0.87	0.83 to 0.90
Superior-nasal	531.57 ± 24.35	18.55	36.36	51.38	1.97	0.89	0.86 to 0.93
Center	472.18 ± 45.41	19.84	38.89	54.96	1.73	0.94	0.92 to 0.95

CV, coefficient of variation; ICC, intraclass correlation coefficient; CI, confidence interval

**Table 3:** Measurements in Group B: Eyes with a history of prior crosslinking.

Sector	Mean ± SD (μm)	Within-Subject SD	Precision	Repeatability	CV (%)	ICC	ICC 95% CI
**Epithelial thickness**							
Superior	56.80 ± 6.39	4.50	8.99	12.72	5.95	0.83	0.77 to 0.87
Superior-temporal	55.27 ± 14.57	23.83	46.71	66.01	4.98	0.89	0.86 to 0.92
Temporal	50.37 ± 5.87	3.09	6.05	8.55	4.75	0.91	0.88 to 0.93
Inferior-temporal	50.63 ± 5.52	3.38	6.62	9.35	4.91	0.88	0.83 to 0.91
Inferior	52.43 ± 5.90	2.84	5.57	7.87	4.16	0.92	0.89 to 0.94
Inferior-nasal	54.09 ± 5.38	2.73	5.34	7.55	4.06	0.92	0.89 to 0.94
Nasal	55.02 ± 5.56	2.91	5.70	8.06	4.39	0.91	0.88 to 0.93
Superior-nasal	56.15 ± 5.73	4.25	8.33	11.77	5.46	0.82	0.75 to 0.87
Center	51.95± 5.92	2.52	4.94	6.99	3.28	0.94	0.92 to 0.96
**Corneal thickness**							
Superior	528.28±42.87	30.34	59.46	84.03	2.61	0.83	0.78 to 0.88
Superior-temporal	506.96 ± 38.78	14.97	29.35	41.48	1.99	0.95	0.93 to 0.96
Temporal	473.61 ± 39.76	20.28	39.76	56.18	1.88	0.91	0.88 to 0.94
Inferior-temporal	462.92 ± 39.60	12.26	24.03	33.96	1.60	0.97	0.96 to 0.98
Inferior	479.37± 40.89	10.17	19.94	28.17	1.66	0.98	0.97 to 0.99
Inferior-nasal	502.87 ± 38.71	12.64	24.78	35.02	1.72	0.96	0.95 to 0.97
Nasal	517.19±39.61	26.43	51.80	73.20	2.29	0.85	0.80 to 0.89
Superior-nasal	531.64 ±38.21	20.98	41.12	58.11	1.91	0.90	0.87 to 0.93
Center	465.11 ±42.28	11.11	21.78	30.78	1.50	0.98	0.97 to 0.98

CV, coefficient of variation; ICC, intraclass correlation coefficient; CI, confidence interval

### CT repeatability

ICC of CT measurements was above 0.82 for both groups. ICC was comparable between the groups, and there was no pattern in the area of lower or higher ICC. Tables 2 and 3 outline CT measurements and repeatability indices in Groups A and B respectively.

### ET repeatability

ICC was above 0.82 in all ET measurements for both groups. ICC was lowest at the superior-nasal aspect and highest at the central aspect for both groups. Tables 2 and 3 outline ET measurements and repeatability indices in Groups A and B respectively.

### Effect of crosslinking on repeatability

There was no statistically significant difference in the repeatability of CT or ET measurements between groups A and B (*p*>0.05).

### Effect of disease severity on repeatability

#### Group A

Keratoconus stage did not significantly affect the CT repeatability in any region. ET measurements were significantly different in the inferior-nasal and central regions. In the inferior-nasal region, eyes with stage IV keratoconus had significantly lower variation in measurements compared to stage I (mean difference = -1.15μm, *p* = 0.03). Similarly, significantly lower variation was found in stage III compared to stage II (mean difference = -0.92μm, *p* = 0.04).

#### Group B

In Group B, there was no statistically significant difference in measurement variation in CT or ET between different stages of keratoconus.

## Discussion

Accurate CT and ET measurements are becoming increasingly important in ectasia diagnosis, monitoring disease progression as well as refractive surgery planning [4]. Abnormal ET profiles are useful for the early detection of keratoconus, as this “doughnut pattern” of localized conical thinning with adjacent thickening becomes increasingly prominent as the disease progresses [10]. This study sought to investigate the repeatability of CT and ET measurements in patients with keratoconus with or without a history of contact lens use or CXL.

There is vast clinical significance in the accuracy and repeatability of epithelial and corneal thickness measurements. The ability of OCT to diagnose early keratoconus otherwise not detectable on clinical and tomographic evaluation may assist in expediting early treatment and preventing disease progression [20]. This can potentially mitigate delays that can occur due to the high need for CXL in certain populations [5]. Epithelial changes on OCT are thought to be the first detectable sign of ectasia, representing an initial epithelial compensation in response to underlying stromal changes [10]. Detection of forme fruste keratoconus is critical in refractive surgery screening, as laser ablation in this patient population can result in suboptimal refractive outcomes and worsening of ectasia [21]. Moreover, CT measurements are instrumental in the preoperative planning of patients undergoing CXL, as certain CXL protocols are contraindicated when TCT is less than 375μm [3]. Repeatable ET measurements are also helpful when contemplating combined treatments with CXL and therapeutic laser ablations in patients with ectasia, as they often have an irregular corneal epithelial thickness profile [3, 22].

CT and ET measurements have previously been shown to be highly repeatable in normal eyes using various OCT devices [23–25]. Although the effect of keratoconus and CXL on repeatability is not well reported, it is generally thought that CXL would impose a lower degree of repeatability compared to normal eyes [9, 26]. A previous study using the Copernicus HR FD-OCT in normal eyes showed a pattern of lower epithelial thickness repeatability in the nasal, inferior and superior-temporal regions [24]. This pattern was not observed in eyes with keratoconus in the present study, which is likely due to the pancorneal CT changes in keratoconus, with the peripheral changes being less pronounced compared to the central cornea [27].

The repeatability of REVO-NX CCT measurements in this study was similar to that of other measurement devices, with the AS-OCT ICC similar to Scheimpflug imaging, scanning slit topography, and SS-OCT [14]. Repeatability values were comparable in both Group A and Group B, indicating that REVO-NX is capable of generating repeatable measurements regardless of a history of crosslinking.

Several studies have reported lower CT repeatability in the peripheral cornea compared to the central cornea using optical, ultrasound, and Visante OCT devices [28, 29]. Dutta *et al*. speculated that this is likely due to the increase in thickness towards the limbus which generates more variable measurements [28]. In the present study, there was no clear pattern of lower repeatability in the peripheral corneal measurements, which suggests that the REVO-NX AS-OCT generates more consistent peripheral corneal and epithelial thickness measurements. However, the ICC values of central and peripheral CT and ET using REVO-NX were similar when compared to SD-OCT and AS-OCT coupled with placido disc technology [29, 30]. Therefore, whilst the REVO-NX generated more consistent measurements across the central and peripheral cornea, the repeatability of measurements compared to other devices should be directly compared and investigated in future studies.

In normal eyes, Visante OCT and RT-Vue OCT produce repeatable CCT and ET measurements in both the central and peripheral cornea, with an ICC reported to be the lowest in the inferior-temporal region [25, 31]. The repeatability of CT and ET measurements have previously been shown to be lower in eyes with keratoconus compared to normal eyes, dry eyes, and eyes with prior refractive surgery using iVue SD-OCT [9]. However, this was not seen in the present study, where the ICC values of CCT and ET were similar to previously reported values in normal eyes [25, 31]. This suggests that keratoconus may not have an impact on the repeatability of measurements using REVO-NX, however, future studies encompassing a normal control group are necessary to support this claim.

SD-OCT is sensitive in detecting the epithelial and corneal remodeling induced by accelerated CXL [26]. Using Optovue RTVue-OCT, Haberman *et al*. reported regionally predictable ET thinning in the inferior and nasal cornea by 1.1–3.2μm at 12 months following CXL [26]. Haberman *et al*. reported that the epithelium in the CXL group is thinner in the temporal, nasal, and inferior aspects compared to non-crosslinked eyes [26]. Corneal haze following CXL has been reported to reduce the repeatability of CCT measurements when using Bausch and Lomb Orbscan [32]. The results of this study suggest that CXL does not have a statistically significant impact on measurement repeatability with the REVO-NX AS-OCT, indicating that it may be a more ideal device when measuring ET and CT in eyes with a history of prior CXL.

The effect of disease severity on the repeatability of CT and ET measurements was also investigated in this study. In Group A, central ET measurement repeatability was lower in more severe disease, which was not observed in Group B. However, in the inferior nasal region, the repeatability was better in higher stages of disease severity, which could be due to the inherently thinner nature of the epithelium in this region in more advanced disease [2, 10, 33], as reduced repeatability has previously been speculated to be associated with an increased thickness profile [28]. Lu *et al*. reported similar findings using RTVue-XR SD-OCT, where the CV values were higher in advanced keratoconus for both ET and CT measurements [34]. For the present study, it is important to note the potential confounding factor of the repeatability and accuracy of the Pentacam device, which was used to determine the severity of keratoconus using the TKC grading system [35].

There are several limitations to this study, including a modest sample size and the lack of a normal control group. Moreover, whilst all measurements were completed between 3 to 6 months post-CXL, this study did not elucidate the temporal relationship between CXL and ET. It may be of value to investigate this longitudinally, where the patients of Group A are recalled for repeat measurements following CXL. Furthermore, contact lens wearers were only instructed to remove their contact lenses 24 hours prior to scanning to minimize inconvenience and maximize participation for those who routinely wear contacts to achieve functional vision. Moreover, some studies demonstrate that soft contact lens users, which were the majority in our cohort, do not exhibit significant alterations in corneal shape [36]. In addition, a histopathological study showed that the duration of contact lens wear did not affect central epithelial pattern types in patients with keratoconus [37]. Finally, future studies investigating any inter-observer impact on repeatability may be useful to ascertain whether the accuracy of measurements is operator-dependent.

In conclusion, the REVO NX is a new spectral domain-OCT device that provides repeatable ET and CT measurements in the central and peripheral cornea in patients and measurements are not affected by a history of CXL. This device is therefore a useful tool for the early diagnosis of keratoconus and monitoring disease progression.

## References

[1] Krachmer JH, Feder RS, Belin MW. Keratoconus and related noninflammatory corneal thinning disorders. Surv Ophthalmol. 1984;28(4):293–322. doi: 10.1016/0039-6257(84)90094-8 6230745

[2] Silverman RH, Urs R, Roychoudhury A, Archer TJ, Gobbe M, Reinstein DZ. Epithelial remodeling as basis for machine-based identification of keratoconus. Invest Ophthalmol Vis Sci. 2014;55(3):1580–7. doi: 10.1167/iovs.13-12578 24557351 PMC3954156

[3] Ziaei M, Gokul A, Vellara H, Meyer J, Patel D, McGhee CNJ. Prospective two-year study of clinical outcomes following epithelium-off pulsed versus continuous accelerated corneal crosslinking for keratoconus. Clin Exp Ophthalmol. 2019;47(8):980–6. doi: 10.1111/ceo.13567 31170327

[4] Ziaei M, Barsam A, Shamie N, Vroman D, Kim T, Donnenfeld ED, et al. Reshaping procedures for the surgical management of corneal ectasia. J Cataract Refract Surg. 2015;41(4):842–72. doi: 10.1016/j.jcrs.2015.03.010 25840308

[5] Goh YW, Gokul A, Yadegarfar ME, Vellara H, Shew W, Patel D, et al. Prospective Clinical Study of Keratoconus Progression in Patients Awaiting Corneal Cross-linking. Cornea. 2020. doi: 10.1097/ICO.0000000000002376 32482959

[6] Ziaei M, Gokul A, Vellara H, Meyer J, Patel D, McGhee CN. Prospective two-year study of clinical outcomes following epithelium-off pulsed versus continuous accelerated corneal crosslinking for keratoconus. Clin Exp Ophthalmol. 2019. doi: 10.1111/ceo.13567 31170327

[7] Ziaei M, Vellara H, Gokul A, Patel D, McGhee CNJ. Prospective 2-year study of accelerated pulsed transepithelial corneal crosslinking outcomes for Keratoconus. Eye (Lond). 2019. doi: 10.1038/s41433-019-0502-3 31273313 PMC7002515

[8] Lian Y, Shen M, Jiang J, Mao X, Lu P, Zhu D, et al. Vertical and horizontal thickness profiles of the corneal epithelium and Bowman’s layer after orthokeratology. Invest Ophthalmol Vis Sci. 2013;54(1):691–6. doi: 10.1167/iovs.12-10263 23221070

[9] Sella R, Zangwill LM, Weinreb RN, Afshari NA. Repeatability and Reproducibility of Corneal Epithelial Thickness Mapping With Spectral-Domain Optical Coherence Tomography in Normal and Diseased Cornea Eyes. Am J Ophthalmol. 2019;197:88–97. doi: 10.1016/j.ajo.2018.09.008 30240724

[10] Reinstein DZ, Gobbe M, Archer TJ, Silverman RH, Coleman DJ. Epithelial, stromal, and total corneal thickness in keratoconus: three-dimensional display with artemis very-high frequency digital ultrasound. J Refract Surg. 2010;26(4):259–71. doi: 10.3928/1081597X-20100218-01 20415322 PMC3655809

[11] Kanellopoulos AJ, Asimellis G. OCT corneal epithelial topographic asymmetry as a sensitive diagnostic tool for early and advancing keratoconus. Clin Ophthalmol. 2014;8:2277–87. doi: 10.2147/OPTH.S67902 25429197 PMC4242699

[12] Han SB, Liu YC, Noriega KM, Mehta JS. Applications of Anterior Segment Optical Coherence Tomography in Cornea and Ocular Surface Diseases. J Ophthalmol. 2016;2016:4971572. doi: 10.1155/2016/4971572 27721988 PMC5046038

[13] Kanclerz P, Hoffer KJ, Rozema JJ, Przewłócka K, Savini G. Repeatability and reproducibility of optical biometry implemented in a new optical coherence tomographer and comparison with a optical low-coherence reflectometer. Journal of Cataract & Refractive Surgery. 2019;45(11):1619–24. 31706516 10.1016/j.jcrs.2019.07.002

[14] Kumar M, Shetty R, Jayadev C, Dutta D. Comparability and repeatability of pachymetry in keratoconus using four noncontact techniques. Indian journal of ophthalmology. 2015;63(9):722–7. doi: 10.4103/0301-4738.170987 26632128 PMC4705708

[15] Dienes L, Kránitz K, Juhász E, Gyenes A, Takács A, Miháltz K, et al. Evaluation of intereye corneal asymmetry in patients with keratoconus. A scheimpflug imaging study. PLoS One. 2014;9(10):e108882. doi: 10.1371/journal.pone.0108882 25296183 PMC4189959

[16] Naderan M, Shoar S, Kamaleddin MA, Rajabi MT. Comparison of corneal measurements in keratoconic eyes using rotating Scheimpflug camera and scanning-slit topography. Int J Ophthalmol. 2015;8(2):275–80. doi: 10.3980/j.issn.2222-3959.2015.02.11 25938040 PMC4413582

[17] Goebels S, Eppig T, Wagenpfeil S, Cayless A, Seitz B, Langenbucher A. Staging of keratoconus indices regarding tomography, topography, and biomechanical measurements. Am J Ophthalmol. 2015;159(4):733–8. doi: 10.1016/j.ajo.2015.01.014 25634534

[18] Feng Y, Varikooty J, Simpson TL. Diurnal variation of corneal and corneal epithelial thickness measured using optical coherence tomography. Cornea. 2001;20(5):480–3. doi: 10.1097/00003226-200107000-00008 11413402

[19] Bland JM, Altman DG. Measurement error. Bmj. 1996;312(7047):1654. doi: 10.1136/bmj.312.7047.1654 8664723 PMC2351401

[20] Temstet C, Sandali O, Bouheraoua N, Hamiche T, Galan A, El Sanharawi M, et al. Corneal epithelial thickness mapping using Fourier-domain optical coherence tomography for detection of form fruste keratoconus. J Cataract Refract Surg. 2015;41(4):812–20. doi: 10.1016/j.jcrs.2014.06.043 25840306

[21] Dupps WJ Jr. Corneal refractive surgery in keratoconus. J Cataract Refract Surg. 46. United States2020. p. 495–6. doi: 10.1097/j.jcrs.0000000000000174 32271291

[22] Ziaei M, Gokul A, Vellara H, Lu LM, Patel DV, McGhee CNJ. Measurement of In Vivo Biomechanical Changes Attributable to Epithelial Removal in Keratoconus Using a Noncontact Tonometer. Cornea. 2020. doi: 10.1097/ICO.0000000000002344 32355111

[23] Prakash G, Agarwal A, Jacob S, Kumar DA, Banerjee R. Comparison of fourier-domain and time-domain optical coherence tomography for assessment of corneal thickness and intersession repeatability. Am J Ophthalmol. 2009;148(2):282–90.e2. doi: 10.1016/j.ajo.2009.03.012 19442961

[24] Vidal S, Viqueira V, Mas D, Domenech B. Repeatability and reproducibility of corneal thickness using SOCT Copernicus HR. Clin Exp Optom. 2013;96(3):278–85. doi: 10.1111/cxo.12002 23316867

[25] Hashmani N, Hashmani S, Saad CM. Wide Corneal Epithelial Mapping Using an Optical Coherence Tomography. Invest Ophthalmol Vis Sci. 2018;59(3):1652–8. doi: 10.1167/iovs.17-23717 29625491

[26] Haberman ID, Lang PZ, Broncano AF, Kim SW, Hafezi F, Randleman JB. Epithelial remodeling after corneal crosslinking using higher fluence and accelerated treatment time. J Cataract Refract Surg. 2018;44(3):306–12. doi: 10.1016/j.jcrs.2017.12.021 29610026

[27] Mathew JH, Goosey JD, Bergmanson JP. Quantified histopathology of the keratoconic cornea. Optom Vis Sci. 2011;88(8):988–97. doi: 10.1097/OPX.0b013e31821ffbd4 21623252 PMC3143234

[28] Dutta D, Rao HL, Addepalli UK, Vaddavalli PK. Corneal thickness in keratoconus: comparing optical, ultrasound, and optical coherence tomography pachymetry. Ophthalmology. 2013;120(3):457–63. doi: 10.1016/j.ophtha.2012.08.036 23177363

[29] Xu Z, Chen S, Yang C, Huang S, Shen M, Wang Y. Reliability of Entire Corneal Thickness Mapping in Normal Post-Laser in situ Keratomileusis and Keratoconus Eyes Using Long Scan Depth Spectral Domain Optical Coherence Tomography. Ophthalmic Res. 2018;59(3):115–25. doi: 10.1159/000478985 28848137

[30] Vega-Estrada A, Mimouni M, Espla E, Alio Del Barrio J, Alio JL. Corneal Epithelial Thickness Intrasubject Repeatability and its Relation With Visual Limitation in Keratoconus. Am J Ophthalmol. 2019;200:255–62. doi: 10.1016/j.ajo.2019.01.015 30689987

[31] Viswanathan D, Kumar NL, Males JJ, Graham SL. Comparative analysis of corneal measurements obtained from a Scheimpflug camera and an integrated Placido-optical coherence tomography device in normal and keratoconic eyes. Acta Ophthalmol. 2015;93(6):e488–94. doi: 10.1111/aos.12622 25495530

[32] Shetty R, Agrawal A, Deshmukh R, Kaweri L, Rao HL, Nagaraja H, et al. Effect of post crosslinking haze on the repeatability of Scheimpflug-based and slit-scanning imaging devices. Indian J Ophthalmol. 2017;65(4):305–10. doi: 10.4103/ijo.IJO_690_16 28513495 PMC5452583

[33] Ziaei M, Meyer J, Gokul A, Vellara H, McGhee CNJ. Direct measurement of anterior corneal curvature changes attributable to epithelial removal in keratoconus. J Cataract Refract Surg. 2018;44(1):71–7. doi: 10.1016/j.jcrs.2017.10.044 29502621

[34] Lu NJ, Chen D, Cui LL, Wang L, Chen SH, Wang QM. Repeatability of Cornea and Sublayer Thickness Measurements Using Optical Coherence Tomography in Corneas of Anomalous Refractive Status. J Refract Surg. 2019;35(9):600–5. doi: 10.3928/1081597X-20190806-03 31498418

[35] Hashemi H, Mehravaran S, Asgari S. The effect of corneal cross-linking on the anterior and posterior parameters of the cornea: A prospective repeatability study. Rom J Ophthalmol. 2019;63(1):68–74. 31198900 PMC6531775

[36] Goudie C, Tatham A, Davies R, Sifton A, Wright M. The effect of the timing of the cessation of contact lens use on the results of biometry. Eye (Lond). 2018;32(6):1048–54. doi: 10.1038/s41433-018-0019-1 29391575 PMC5997671

[37] Sorbara L, Lopez JCL, Gorbet M, Bizheva K, Lamarca JM, Pastor JC, et al. Impact of contact lens wear on epithelial alterations in keratoconus. J Optom. 2021;14(1):37–43. doi: 10.1016/j.optom.2020.02.005 32376120 PMC7752984

